# Inter-Girdle Coordination as a Predictive Measure of Low Back Pain

**DOI:** 10.3390/s25164928

**Published:** 2025-08-09

**Authors:** Paul Pascaud, Nicolas Houel, Philippe Dedieu

**Affiliations:** 1 Clinic of Physiotherapy, 24000 Perigueux, France; 2 EA 7507, PSMS, UFR-STAPS, University of Reims Champagne-Ardennes, 51100 Reims, France; nicolas.houel@univ-reims.fr

**Keywords:** low back pain, dynamic pattern theory, prevention, gait

## Abstract

**Highlights:**

**What are the main findings?**

- Inter-girdle coordination is a relevant determinant of LBP potential risk.
- Inter-girdle coordination allows trunk stability during gait.

**What is the implication of the main findings?**

- Range of motion in the transverse plane is relevant in functional prevention of LBP.

**Abstract:**

Low back pain (LBP) is a common chronic musculoskeletal disorder with significant interpersonal variability, so individual treatment strategies are difficult to implement. Prevention of recurrence, particularly through exercise and physical activity, appears to be necessary to avoid acute episodes. The present study aims to find whether some behavioral characteristics (particularly inter-girdle coordination) in the painless period in patients who had experienced LBP could be detected as relevant to prevent acute recurrences. Thirty-four young adults participated in the study. They were recruited from outpatient physiotherapy clinics. Sixteen subjects formed the Control group (CG), and eighteen subjects formed the patient group (LBPG), with no differences between groups in individual parameters. Moreover, the Duke Health Profile (General Health Score, Perceived Health Score, and Pain Score) was calculated and did not show differences between groups. Kinematic data, muscular activity, and inter-girdle coordination were captured. Our results show significant differences between groups only for inter-girdle coordination, which is less out-of-phase in the LBP group. The emergence of coordinative patterns (in the present work, the inter-girdle coordination) expresses behavior in an integrative way and allows the prevention of acute recurrences despite a lack of differences in usual gait parameters.

## 1. Introduction

Low back pain (LBP) is a common chronic musculoskeletal disorder [1]. Given the difficulties in identifying the cause of LBP [2] and the interpersonally variable course of LBP [3], individual treatment strategies are difficult to implement. Therefore, strategies for the prevention of recurrence appear to be effective in preventing acute episodes [4], particularly the use of exercise and physical activity [5,6].

An alteration in the strategy for lumbopelvic stabilization had been described as responsible for low back pain (LBP) and pelvic girdle pain (PGP) [7,8]. Although different core-stabilization approaches exist, the evidence is controversial [9]. Moreover, results need to be cautiously interpreted due to the lack of original studies [10].

Whereas walking has been shown as a common physical activity with numerous health benefits (in an isolated or associated manner, for example, with gardening) [11], gait analysis in different individual situations showed modifications in the inter-girdle coordination, particularly in the transverse plane. Lack of coordination of pelvis and thorax rotations had been described to be predominant in LBP patients when the walking velocity increases [12]. This phenomenon can be defined as a tendency for pelvic and scapular girdle rotations to occur in the same direction. This phenomenon is referred to as in-phase coordination tendency [12]. On the contrary, the same results were described when the walking speed decreases in normal subjects without pain [13]. Furthermore, in the face of varying constraints, the inter-girdle relative timing was identified as the most pertinent descriptor for characterizing the adaptive properties of the locomotor system [14].

The present study hypothesizes that inter-girdle coordination is a relevant and sensitive characteristic of gait adaptability in the face of various constraints. This coordination may serve as a preventative function in identifying asymptomatic low back pain (LBP) patients. Consequently, in subsequent instances, the employment of functional therapeutics in the transverse plane may prove advantageous. The prevailing tendency would be toward functional stabilization of the trunk and pelvic movements [15,16]. However, promoting the mobility of the girdles in the transverse plane and their independence would favor the adaptability of movement in ecological conditions [14].

The aim of the present study is to explore whether inter-girdle coordination could preventively identify asymptomatic LBP patients while walking.

## 2. Materials and Methods

This study was conducted in accordance with the Helsinki Declaration and has been approved by the local ethics committee.

### 2.1. Participants

Thirty-four young adults participated in the study (Table 1). They were recruited from outpatient physiotherapy clinics. Sixteen subjects formed the Control group (CG). They were in good health with no history of back pain or leg pain that might be attributed to the back within the last 12 months. Eighteen subjects formed the patient group (LBPG). They suffered with low back pain (i.e., pain over the L1-sacrum region without any radiation to areas distal to the gluteal crease) but had no restriction in lumbar spine movement. They were diagnosed by a physician in accordance with criteria used by Vogt et al. [17]. At the time of experimentation, they had not experienced pain incidents for one year at least. The sample size was estimated based on the findings of a previous study that investigated disparities in inter-girdle coordination during locomotion [14].

Participants were informed about the experimental procedure, methods, how their data will be used, and the benefits and possible risks involved in the study prior to the attainment of written consent. They volunteered to participate in the study and agreed to the publication. All participants were fully informed that their anonymity is assured. The key for data anonymization is secured by the principal investigator.

At the time of data collection, all individuals in the LBP group were in symptom remission (defined as a score of less than 0.5/10 cm on a visual analog scale for current pain) in accordance with Smith and Kullig [18]. Disability due to LBP was quantified using the French version of the Roland–Morris Disability Questionnaire (RMDQ), which is widely used and recommended in the management of chronic LBP [19,20,21].

All the participants answered the Duke Health Profile [22]. The General Health Score, Perceived Health Score, and Pain Score were calculated.

### 2.2. Procedure

Participants were invited to walk barefoot at a spontaneous speed back and forth in the Gait Analysis Room (10 m × 4.5 m). The decision to employ a spontaneous speed was motivated by the objective of minimizing the necessity for gait modifications due to the imposed speed constraint [23].

In accordance with Dedieu et al. [24], approximately one minute following the walking onset, the recording procedure was initiated while the participants were walking in a straight line. A total of five successive gait cycles were extracted and included in the analysis.

Each participant performed one single trial.

The 3D coordinates of markers placed on body landmarks were recorded using a motion analysis system (Codamotion™, Charnwood Dynamics Ltd., Rothley, UK) in accordance with the Plug-in-Gait Marker placement.

Muscular activity was recorded through a surface EMG system (Trigno Wireless System™, Base Station Trigno SP-W02, 16 channels, wireless electrodes Trigno SP-W01 (center-to-center inter-electrode distance of 2 cm), Delsys, Boston, MA, USA) with a sampling rate of 2000 Hz. In accordance with a previous study [25], the tibialis anterior, soleus, gluteus maximus, and lumbar part of the erector spinae (iliocostalis) were selected. It was possible to understand direct or indirect muscular activity around the lower back.

Following appropriate skin preparation [26], electrodes were placed on both sides over the bellies of the tibialis anterior (at 1/3 on the line between the tip of the fibula and the tip of the medial malleolus), soleus (at 2/3 of the line between the medial condylis of the femur to the medial malleolus), rectus femoris (at 50% on the line from the anterior spina iliaca superior to the superior part of the patella), gluteus maximus (at 50% on the line between the sacral vertebrae and the greater trochanter), and erector spinae (iliocostalis) (one finger width medial from the line from the posterior spina iliaca superior to the lowest point of the lower rib, at the level of L2) in accordance with the SENIAM recommendations for sensor locations.

### 2.3. Data Processing

The raw 3D coordinates were smoothed through a two-way, first-order Butterworth low-pass filter with a cutoff at 6 Hz [27].

The gait cycle duration was calculated as the time between two successive contacts of the same foot determined by a foot switch fixed on the plantar face of the heel and the first and fifth metatarsal heads. The average ratio between the stance duration and the gait cycle duration was then computed for each gait cycle and expressed as a percentage of the total cycle duration.

Following Dedieu et al. [24], the kinematic data of the ankle, knee, and hip as well as the pelvic tilt in the sagittal plane were calculated from the 3D coordinates and spatially normalized within the range of −1 to1, and the time was normalized, so that each gait cycle lasted 100 samples [28].

As a pertinent measure of inter-girdle coordination, the angle formed by the scapular and pelvic girdles in the horizontal plane was computed between the line that connected the two markers of the shoulders and the one that connected the two anterior superior iliac spines [14]. The relative phase value between the motion of the scapular and pelvic girdles was assessed by a Continuous Relative Phase (CRP) algorithm, using a Hilbert transform within the range of 0° ≤ CRP ≤ 180° [29].

The EMG signal was band-pass filtered between 10 and 400 Hz. The linear envelope was obtained by second-order, two-way Butterworth filtering of the rectified signals at 6 Hz [30,31]. Each linear envelope was normalized in time over 100 samples and in magnitude in reference to the highest peak of each gait cycle [31,32]. A muscle was considered to be active when the signal magnitude was higher than the magnitude of two standard deviations computed during relaxed upright standing [33]. The start and duration of muscle activity were expressed as a percentage of the gait cycle.

### 2.4. Statistical Analysis

Differences in inter-girdle CRP between groups were compared using *t*-tests.

The mean values for the start, end, and duration of muscle activity were compared using *t*-tests.

A k.Means Clustering Analysis was carried out using an average linkage method for CRP.

Effect sizes were calculated using Cohen’s d, with 0.8 indicating a large effect size, 0.5 a medium effect size, and 0.2 a small effect size [34,35].

The significance level for all analyses was set at *p* < 0.05.

## 3. Results

### 3.1. Individual Parameters

Individual parameters did not show any differences between groups. One can note that the results of the Duke Health Profile (in the three aspects evaluated) do not allow discrimination between the Control and LBP groups in the same way that the Roland–Morris Disability Questionnaire does (Table 1).

### 3.2. Gait Parameters and Normalized Joint Angles

The ratio between the stance period and the gait cycle duration was not significantly different between both groups (Table 2).

The results did not indicate significant differences in the mean of gait parameters and in the mean angle of the ankle, the knee, or the hip as well as pelvic tilt in the sagittal plane between both groups (Table 2).

### 3.3. Inter-Girdle Coordination

The Continuous Relative Phase (CRP) is a relevant measure of inter-girdle coordination. An absolute value between 90° and 180° indicates a tendency of out-of-phase coordination, that is, an opposite direction between the pelvic and the scapular girdle along the gait cycle. The mean CRP between Control and LBP groups indicates a significant difference. In accordance with previous works [36], CRP is closer to 180° in the CG than in the LBPG (132.06° (SD: 9.86) vs. 109.8° (SD: 9.7); *p* = 0.001; *d* = 1.49); this indicates a more out-of-phase coordination in non-pathologic participants. In summary, inter-girdle dissociation appears to be higher in the CG relative to that in the LBPG, signifying greater independence in motion between the pelvic and scapular girdles in the transverse plane (Table 2).

### 3.4. Muscular Pattern

**Figure 1:**
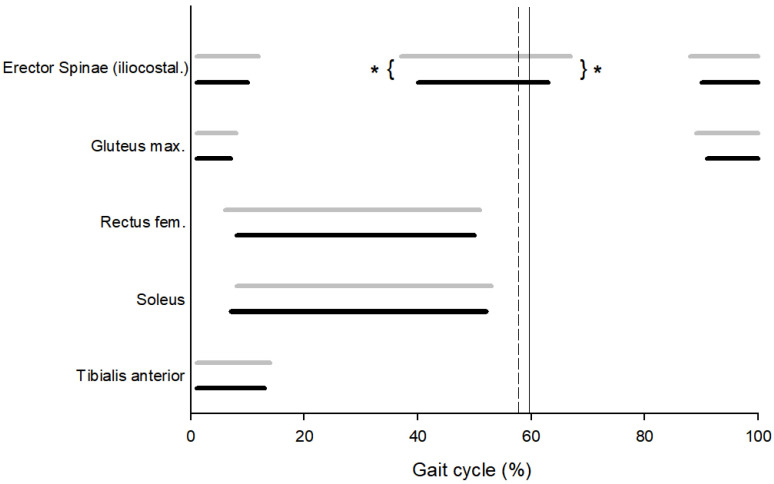
Start and duration of muscular activity during gait cycle in Control group (black line) and Low Back Pain group (gray line) (from top to bottom: erector spinae (iliocostalis), gluteus maximus, soleus, and tibialis anterior). The vertical line indicates the mean value of toe-off for the Control group (solid line) and the LBP group (dashed line); (* denotes significant differences).

The onset and duration of the activity of the tibialis anterior, soleus, rectus femoris, and gluteus maximus muscles do not vary among the study’s participant groups. However, in the LBPG, the erector spinae (iliocostalis) are activated earlier than in the CG (40.69% (SD: 4.69) vs. 37.44% (SD: 3.48); *p* = 0.001; *d* = 0.76) and remain activated longer in the LBPG than in the CG (30.75% (SD:2.08) vs. 23.75% (SD: 1.84): *p* = 0.02; *d* = −1.72). Only the erector spinae (iliocostalis) activity highlights a difference between the Control and LBP groups with an early start and a longer duration of muscular activation in the LBPG, indicating an anticipated and longer activity before the end of the stance phase and the beginning of the oscillation phase. This so-called pre-swing phase is characteristic of the mass transfer from the side currently on stage (and that will take off) to the side that is just starting its ground contact (Figure 1).

### 3.5. k.Means Clustering Analysis

**Figure 2:**
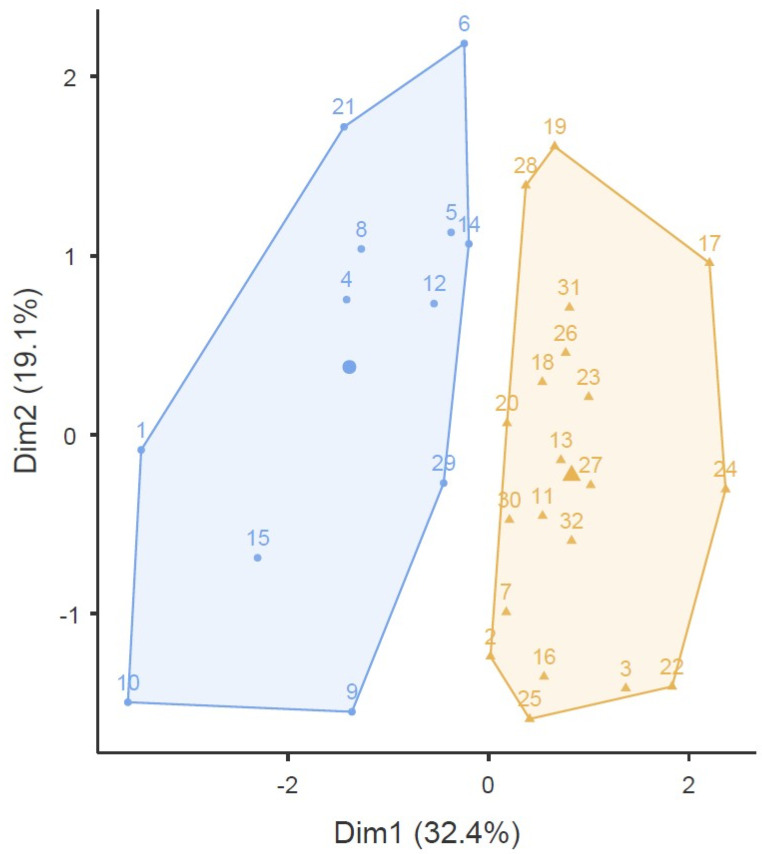
Cluster plot.

The k.Means Clustering Analysis using an average linkage method for CRP identifies two clusters. The correlation analysis between clusters and experimental conditions showed a significant correlation (ρ = 0.78) (Figure 2).

## 4. Discussion

The objective of the present study was to determine whether inter-girdle coordination could serve as a preventative tool for identifying asymptomatic (LBP) patients with a documented medical history of LBP while walking. The subjects participating in this study did not exhibit any symptoms of LBP at the time of the experiment. The sole LBPG participant had a documented medical history of LBP.

### 4.1. Main Findings

The primary outcome of the present study indicates that the inter-girdle coordination is the main discriminant between CG and LBPG participants, in addition to erector spinae (iliocostalis) activity. The remaining study variables did not demonstrate differences between the groups.

### 4.2. Comparison with the Literature

Despite the substantial variations in spatio-temporal parameters reported by Vogt et al. [17], which indicate that individuals suffering from back pain exhibit a range-of-motion limitation due to pain when compared with healthy controls for hip joint range of motion and stride time while walking, the present study found no significant differences. The absence of acute lower back pain (LBP) among study participants at the time of experimentation offers a compelling explanation for the observed outcomes. The absence of observable differences in lower limb joint range of motion, irrespective of the specific joint in question (i.e., the ankle, knee, or hip), is of interest.

Moreover, pelvic tilt in the sagittal plane and spatio-temporal parameters could not differentiate between the Control and LBP groups. It could be supposed that LBP history does not imply an ROM limitation in the painless condition but a functional limitation in the acute situation only [17]. Lack of pain at the time of the experiment could explain why the gait analysis parameters do not seem to be affected by the LBP history. Similarly, the absence of pain seems to allow postural control of trunk sway while walking, particularly during hip loading/unloading strategies in the pre-swing phase [37]. Indeed, this aspect seems crucial, as noted by delle Volte et al. [38], and appears to characterize the gait of patients suffering from LBP. Participants in our study were physically active and did not receive any functional care at the time of the experiment. They did not perceive any physical constraints related to their LBP (for the LBP group) in accordance with the Roland–Morris Disability Questionnaire. It is not feasible to assume that they were habituated to pain, particularly pain that is induced by some form of usual functional constraint [39].

The lack of difference in trunk postural sway between both Control and LBP groups could be explained by muscular pattern differences. The earlier and longer erector spinae muscle activation during pre-swing in the LBP group appears to confirm the need to stabilize the trunk during the body weight transfer to the contralateral side that has just landed [40]. The absence of a significant difference in gluteus maximus muscle activity between the two groups appears to confirm that this stabilization occurs at the trunk level itself and not at the pelvis level. Moreover, because the erector spinae muscle is more axial along the spinal column, the increased muscular activity duration could limit the trunk torsion phenomenon, thus limiting dissociation between both the pelvic and the scapular girdles. This could be linked to the observed differences in inter-girdle coordination.

Despite the apparent homogeneity in lower limb kinematics among all the participants, the LBP group demonstrated reduced out-of-phase inter-girdle coordination, signifying a lower independence in motion between the pelvic and scapular girdles in the transverse plane [14]. Even though variability remains roughly the same, the inter-girdle Continuous Relative Phase is significantly different between both Control and LBP groups. In accordance with previous works [12,14,36], inter-girdle coordinative patterns seem to highlight that locomotor behavior expresses the adaptive properties of the locomotor system even in painless situations in patients with an LBP history. A less out-of-phase inter-girdle coordination pattern reduces the range of dissociation and results in a less adaptive coordination pattern. In this situation, constraints from the LBP history or muscular pattern (particularly differences in erector spinae muscle activity duration) appear to lead to stiffened coordination. Whereas painful periods are far from the day of the experiment, it is difficult to link possible preventive behavior and a history of LBP episodes in the present results. The observed behavior seems to express a spontaneous emergent pattern. Interestingly, inter-girdle coordination appears to be a pertinent and precise variable to discriminate between participants either in a deductive or an inductive direction. These findings indicate that the inter-girdle coordination is modified when a participant has already suffered LBP, but this modification does not lead to an altogether new pattern of coordination. The results of the present study are in agreement with those of Hodge and Tuker [41], where adaptation to pain could lead to redistribution within and between muscles and where adaptation to pain could change behavior with potential long-term consequences.

### 4.3. Clinical Implications

The results are in continuation with those of previous studies supporting the idea that, in the face of changing constraints, the CNS can modify inter-joint coordination while still preserving the same basic synergy [42]. Following previous work on ankle sprain history [20] or in constraints, such as rules, in sports [43], the emergence of coordinative patterns (in the present work, the inter-girdle coordination) expresses behavior in an integrative way. Whereas all participants in the present study consider themselves in good health status, without differences between groups in perceived pain, inter-girdle coordination allows us to precisely discriminate between participants regarding their functional antecedents even in painless periods, independently of kinematic measures. Therefore, coordination in the transverse plane could describe underlying emerging dynamics even if they are not spontaneously grasped. This opens perspectives to prevent possible risk factors or to propose adapted preventive functional activity.

### 4.4. Study Limitations and Future Directions

While the results appear to offer novel insights into the potential for preventing low back pain (LBP) through the promotion of independent movements of the pelvic and scapular girdles in the transverse plane, it is essential to exercise caution and refrain from hasty conclusions. It is noteworthy that the present study was conducted on participants who were asymptomatic at the time of the study and whose number remains limited. Furthermore, the lack of psychological assessments, such as fear-avoidance beliefs and anxiety, hinders a comprehensive understanding of the multifaceted parameters associated with chronic pain, including low back pain (LBP).

The evolution of this study should allow for the comparison of symptomatic subjects with asymptomatic subjects. A similar approach could involve conducting an experiment in which the same variables are studied in the same participants (symptomatic vs. asymptomatic) but with locomotion constraints (e.g., imposed speed, use or not of the upper limbs). Additionally, the walking/running transition speed (as an expression of coordination transition) could be determined.

### 4.5. Conclusions

The interest of this study is to focus on novel perspectives and methodologies for addressing low back pain (LBP) through the examination of behavior and motor control. It presents perspective on the potential for preventing or rehabilitating LBP by mobilizing the back and pelvis three dimensionally, with a particular focus on the independence of girdles in the transverse plane.

## Figures and Tables

**Table 1:** Individual parameters: Mean data are presented with standard deviation in parentheses. *p* is the statistical probability of difference between groups. Statistical significance was accepted at the 5% level for *p*.

	Control Group (*n* = 16)	Low Back Pain Group (*n* = 18)	*p*
Age (years)	29.41 (3.32)	29.67 (2.92)	*0.41*
Height (m)	1.65 (0.09)	1.67 (0.08)	*0.23*
Mass (kg)	62.25 (11.17)	64.25 (7.98)	*0.28*
Body Mass Index	22.73 (2.25)	21.93 (1.98)	*0.39*
Duration from Low Back Pain Diagnostic (months)		27.25 (9.76)	
Disability (Roland–Morris Disability Questionnaire)	0.88 (0.72)	1.94 (1.02)	*0.001*
Duke Health Profile (General Health Score)	82.63 (4.65)	80.81 (5.98)	*0.17*
Duke Health Profile (Perceived Health Score)	87.5 (22.36)	78.13 (22.62)	*0.14*
Duke Health Profile (Pain Score)	75 (31.62)	68.75 (30.96)	*0.29*

**Table 2:** Mean value and standard deviation in parentheses of stance phase and speed, normalized hip range of motion (ROM), normalized knee ROM, normalized ankle ROM, and inter-girdle CRP. Statistical significance indicated with * was accepted at the 5% level for *p; d* indicates the effect size when results are significantly different.

	Control Group (*n* = 16)	Low Back Pain Group (*n* = 18)	*p*	*d*
Stance phase (%)	58.06 (2.62)	59.25 (2.49)	*0.20*	
Speed (m.s^−1^)	0.96 (0.04)	0.97 (0.04)	*0.46*	
Hip (°)	0.26 (0.12)	0.18 (0.16)	*0.09*	
Knee (°)	0.41 (0.01)	0.4 (0.01)	*0.11*	
Ankle (°)	0.49 (0.17)	0.58 (0.14)	*0.12*	
Inter-girdle CRP (°) *	−132.06 (9.86)	−109.88 (9.7)	*0.001*	*1.49*

## Data Availability

The datasets presented in this article are not readily available due to time limitations. Only the summary tables have been retained.
